# Mycobacterial Aerosols and Respiratory Disease

**DOI:** 10.3201/eid0907.02-0415

**Published:** 2003-07

**Authors:** Joseph O. Falkinham

**Affiliations:** * Virginia Polytechnic Institute and State University, Blacksburg, Virginia, USA

**Keywords:** mycobacteria, aerosols, respiratory disease, perspective

## Abstract

Environmental opportunistic mycobacteria, including *Mycobacterium avium*, *M. terrae*, and the new species *M. immunogenum,* have been implicated in outbreaks of hypersensitivity pneumonitis or respiratory problems in a wide variety of settings. One common feature of the outbreaks has been exposure to aerosols. Aerosols have been generated from metalworking fluid during machining and grinding operations as well as from indoor swimming pools, hot tubs, and water-damaged buildings. Environmental opportunistic mycobacteria are present in drinking water, resistant to disinfection, able to provoke inflammatory reactions, and readily aerosolized. In all outbreaks, the water sources of the aerosols were disinfected. Disinfection may select for the predominance and growth of mycobacteria. Therefore, mycobacteria may be responsible, in part, for many outbreaks of hypersensitivity pneumonitis and other respiratory problems in the workplace and home.

Hypersensitivity pneumonitis is an occupational hazard of workers in two different industries, automobile manufacturing (e.g., metal working) and leisure (e.g., indoor swimming pools). Pulmonary illness and infection have also been a consequence of exposure to aerosols generated by hot tubs, spas, and coolant baths. Respiratory problems have also been associated with exposure to water-damaged buildings during reconstruction, and mycobacteria isolated from materials from such buildings have been shown to provoke inflammatory reactions. The outbreaks share the common feature of aerosol exposure and respiratory illness. I propose that exposure to aerosols containing mycobacteria is a common feature of the outbreaks and that mycobacteria or their products could be responsible for the respiratory symptoms.

Epidemiologic studies have established that the workers in such outbreaks were exposed to aerosols generated in the workplace from water that was either a work tool (e.g., metalworking fluid) or an integral part of the workplace or household (e.g., swimming pools and hot tubs) (*1*–*7*). Outbreaks of respiratory disease occurred in spite of disinfectant treatment of the waters or fluids to reduce the number of microorganisms. Living or working in water-damaged buildings or as a consequence of reconstruction of water-damaged buildings has also been associated with outbreaks of respiratory problems (*8*,*9*). Respiratory disease has been associated with mycobacteria in reservoirs, aerosols, or structural material in a number of cases (*2*,*3*,*6*,*7*,*9*).

## Hypersensitivity Pneumonitis in Workers Exposed to Metalworking Fluid

An estimated 1.2 million workers in the United States are exposed to aerosols generated by metal grinding (*10*). Metalworking fluids are widely used in a variety of common industrial metal-grinding operations to lubricate and cool the tool and the working surface. Metalworking fluids are oil-water emulsions that contain paraffins, pine oils, polycyclic aromatic hydrocarbons, and heavy metals (*10*,*11*). Exposure to metalworking fluid aerosols can lead to hypersensitivity pneumonitis and chronic obstructive pulmonary disease (*1*,*6*,*12*–*14*). Mycobacteria were recovered significantly more frequently from metalworking fluid samples collected from facilities where hypersensitivity pneumonitis was found; compared to facilities that did not have hypersensitivity pneumonitis (*6*). In one study, exposure to metalworking fluid mist resulted in hypersensitivity pneumonitis in 10 workers (*7*). Acid-fast microorganisms identified as mycobacteria were present in the reservoir at 10^7^ CFU/mL (*7*). A mycobacteria in the reservoir was considered to be a likely cause of the hypersensitivity pneumonitis because one patient was infected by a *Mycobacterium* sp. and had antibodies against the reservoir fluid (*7*).

Hypersensitivity pneumonitis appeared in spite of disinfection of the metalworking fluid with morpholine, formaldehyde, or quaternary ammonium-based disinfectants (*1*,*6*,*12*,*13*), and mycobacteria were recovered from the metal working fluid (*6*,*14*,*15*). Mycobacteria are resistant to formaldehyde and quaternary ammonium disinfectants (*16*) and the heavy metals in metalworking fluids (*17*). Further, mycobacteria can grow on the organic compounds in metalworking fluid, including the paraffins, pine oils, and polycyclic aromatic hydrocarbons (*18*,*19*) and can degrade the disinfectant morpholine (*20*). Mycobacteria present in the water (*21*) can likely grow on the organic compounds in metalworking fluids in the absence of competitors after disinfection. Cleaning would not be expected to eradicate mycobacteria because of their ability to form biofilms (*21*,*22*). Adding disinfectant and cleaning the reservoir in one facility did not prevent the reappearance of mycobacteria (7 x 10^5^ CFU/mL by 2 weeks [*7*]). Further, disinfectant treatment would likely result in selection of mycobacteria remaining after the cleaning.

## Hypersensitivity Pneumonitis in Swimming Pool Attendants

Granulomatous pneumonitis has been reported in lifeguards (“lifeguard lung”) who worked at an indoor swimming pool that featured waterfalls and sprays (*5*). Affected lifeguards with symptoms worked longer hours than unaffected lifeguards (*5*), which demonstrated a dose-response effect. The waterfalls and sprays increased the number of respirable particles fivefold and the levels of endotoxin eightfold (*5*). Based on the presence of endotoxin in the aerosol samples, endotoxin exposure was suggested as the cause of the pneumonitis in lifeguards (*5*). However, subsequent data provided evidence of a possible second factor resulting in hypersensitivity pneumonitis; aerosols containing mycobacteria were shown to cause granulomatous lung disease (*4*). Others have reported high numbers of mycobacteria in swimming pools and whirlpools (*23*) and in hot tubs (*2*,*3*,*24*). Further, amoebae were reported in the indoor swimming pool where lifeguards reported pneumonitis (*5*). Mycobacteria, including *M. avium* and *M. intracellulare*, can survive and grow in phagocytic amoebae (*25*) and protozoa (*26*). In fact, *M. avium* grown in amoebae or protozoa are more virulent (*25*; Falkinham JO, unpub. data). Mycobacteria are resistant to chlorine (*27*) and preferentially aerosolized from water (*28*).

## Mycobacterial Disease after Exposure to Aerosols Generated by Hot Tubs

Hypersensitivity pneumonitis and mycobacterial pulmonary disease has been reported after exposure to hot tubs (*2*,*3*,*24*). The mycobacteria isolated (e.g., *M. avium*) were likely responsible for the infections based on the identity of patient and hot tub mycobacterial isolates by either restriction fragment length polymorphism analysis (*24*) or multilocus enzyme electrophoresis (*2*,*3*). Further, exposure was followed closely by the onset of symptoms, and the extent of symptoms was related to the length of exposure (i.e., time spent in the hot tub) (*2*,*24*). Although these reports do not document the use of disinfectants in the hot tubs, the waters had been heated. Mycobacteria are relatively resistant to high temperature (*29*) and concentrated in hospital hot water systems (*30*).

## Hypersensitivity Pneumonitis in Occupants of Water-Damaged Buildings

Inflammatory reactions—including eye irritation, respiratory infections, wheeze, bronchitis, and asthma—in workers in water-damaged or “moldy” buildings have been associated with the presence of high numbers of microorganisms (*8*). Mycobacteria were recovered from materials collected from water-damaged buildings, as well as from microorganisms normally associated with building materials (*9*). During reconstruction, those mycobacteria could be aerosolized in the dust. Although other microorganisms could be responsible for the respiratory problems, both saprophytic (e.g., *M. terrae*) and pathogenic (e.g., *M. avium*) strains isolated from moldy buildings were capable of inducing inflammatory responses in a mouse macrophage cell line (*31*). The mycobacteria elicited dose-dependent production of cytokines interleukin-6 and tumor necrosis factor-α, nitric oxide, and reactive oxygen species from the murine macrophage (*31*). Because whole mycobacterial cells were used in the assays (*31*), whether cell metabolites, which are likely easily aerosolized, were responsible for the induction of inflammatory reactions is not known. Heat-shock proteins from a number of mycobacterial species have been shown to generate Th1-type responses, airway inflammation, and airway hyperresponsiveness (*32*). This evidence suggests that mycobacteria or their metabolites are possible causes of respiratory disease in persons exposed to water-damaged buildings.

## Ecology of Mycobacteria

The unique combination of physiologic characteristics that distinguish the environmental opportunistic mycobacteria make them likely agents for causing respiratory disease in these diverse settings. Mycobacteria are found in a great variety of natural and human-influenced aquatic environments, including treated drinking water (*21*) and aerosols (*33*). Mycobacteria in drinking water are associated with the presence of particulates (*21*). Although these microbes are grown in rich media in the laboratory, they are oligotrophic and capable of substantial growth in low concentrations of organic matter. For example, *M. avium* and *M. intracellulare* can grow in natural and drinking water over a temperature range of 10°C to 45°C (*34*). Mycobacteria are relatively resistant to high temperatures. For example, 10% of cells of a strain of *M. avium* survived after 1 h at 55°C (*29*). Mycobacteria are slow growing as a consequence of their fatty acid- and wax-rich impermeable cell wall (*35*). The resulting cell surface hydrophobicity permits adherence to solid substrates (e.g., pipes and leaves) in aquatic environments, which results in mycobacteria’s persistence and resistance to being washed away at high flow rates (*21*,*22*). Further, hydrophobicity is undoubtedly associated with the ability of these bacteria to metabolize a wide variety of nonpolar organic compounds (*18*–*20*) that are constituents of metal working fluids (*15*,*16*).

## Resistance of Mycobacteria to Disinfection

Mycobacteria are very resistant to the disinfectants used in water treatment, including chlorine and ozone (*27*). For example, *M. avium* is almost 500 times more resistant to chlorine than is *Escherichia coli* (*27*). Mycobacteria are also quite resistant to agents used for surface and instrument disinfection, including quaternary ammonium compounds, phenolics, iodophors, and glutaraldehyde (*16*,*22*,*23*,*36*) and can degrade the disinfectant morpholine (*20*). Hydrophobicity and impermeability are undoubtedly factors contributing to the disinfection resistance of mycobacteria (*35*). Chemical or enzymatic removal of surface lipid, while not reducing viability, reduces surface hydrophobicity and alters cell charge (*37*). Because of their inherent impermeability, mycobacteria grow relatively slowly compared to other bacteria. The slow growth is not necessarily a disadvantage because it correlates with increased resistance to antimicrobial agents (*35*), including chlorine (Falkinham JO, unpub data).

Exposure of a mixed microbial population to disinfectants results in selection of a disinfectant-resistant or tolerant population (*38*). The persistence and growth of mycobacteria in drinking water systems (*21*) are due, in part, to their disinfectant-resistance (*27*) and ability to grow under oligotrophic conditions (*21*). Disinfection of swimming pools, therapy pools, and spas or hot tubs with chlorine is expected to kill nonmycobacterial flora and to permit the growth of even the slowly growing mycobacteria in the absence of competitors for nutrients. High temperature would also be expected to result in enrichment of mycobacteria (*29*,*30*). Resistance to disinfectants could also lead to the proliferation of mycobacterial populations in metal working fluid and coolants after disinfection (*6*,*12*,*13*).

## Aerosolization of Mycobacteria

Although *M. tuberculosis* is transmitted between patients through aerosols, little information exists on aerosolization of the environmental opportunistic mycobacteria (e.g., *M. avium* and *M. intracellulare*). Patient-to-patient transmission of environmental opportunistic mycobacteria does not occur (*39*). *M. avium* and *M. intracellulare* are readily aerosolized from aqueous suspension (*28*,*33*). Transfer of mycobacteria occurs as a result of binding of mycobacterial cells to air bubbles and ejection of water droplets after the air bubbles reach the liquid surface (*28*). Aerosolization can result in >1,000-fold increase in numbers of viable mycobacterial cells per milliliter of water droplets ejected from water (*28*). Mycobacteria in natural aerosols are found in particles and droplets (i.e., <5 μm) that can enter the alveoli of the human lung (*28*,*33*). Cell surface hydrophobicity, not surface charge, is a major determination of enrichment in ejected droplets (*28*). Transfer of mycobacteria from water to air is subject to prevailing physiochemical conditions and can be manipulated. Salts (e.g., NaCl) or detergents reduce the rate of transfer of mycobacteria from water to air by ejected droplets. The influence of the components of metalworking fluid or of chlorine or other disinfectants in water upon aerosolization mycobacteria is unknown.

## Mycobacteria and Immune Responses and Airway Inflammation

Mycobacterial cells and cellular components provoke inflammatory responses. Cells of mycobacterial strains isolated from material collected from water-damaged buildings provoke inflammatory responses in macrophages (*31*). Mycobacterial heat-shock proteins generate Th1-type responses, airway inflammation, and hyperresponsiveness (*32*). The mycolic acid-containing glycolipids, mannose-containing phospholipids, glycopeptidolipid mycosides, phenolglycolipid mycosides, and sulfatides that are unique to mycobacteria have all been reported to stimulate immune responses in animals (*40*). Further, mycobacteria produce a variety of extracellular primary and secondary metabolites (*19*) that could be aerosolized and trigger immune responses, including hypersensitivity pneumonitis. Some of these immunostimulatory compounds are produced in response to growth on polycyclic aromatic hydrocarbons (*18*). Unfortunately, the studies of inflammatory responses provoked by mycobacteria have been limited to whole cells grown under a single condition (*31*) or single proteins (*32*). The influence of growth conditions (e.g., growth in metalworking fluid or chlorinated water) or cell fractions (e.g., membranes) or metabolites to stimulate inflammatory responses has not been measured.

## Conclusion

Contemporary reviews of airway dysfunction all describe the need for information concerning microbial agents of workplace and household exposure (*41*). Although many more studies are needed, the evidence points to a role of environmental opportunistic mycobacteria in provoking hypersensitivity pneumonitis, respiratory disease, and respiratory infection in both the workplace and home. In addition to the recovery of identical species and types of mycobacteria from reservoirs and patients, physiologic characteristics of mycobacteria are consistent with their presence in the sources, transmission by means of aerosols, and illnesses. Identifying the factors that influence the presence of mycobacteria in aerosols in these workplaces would have an impact on workers in a variety of occupational settings.

On the basis of several physiologic and ecologic characteristics of mycobacteria, several approaches to reduce the impact of mycobacteria in these settings are possible. Because mycobacteria are associated with particulates (*21*), their numbers in reservoirs can be reduced by removal of particular matter (e.g., filtration). UV light can be used to reduce mycobacterial numbers. Disinfection of mycobacteria at high temperatures (e.g., 40°C) is more effective at reducing numbers, especially if cells were grown at lower temperatures (e.g., 30°C). Agents or combinations with surfactant or detergent-like and disinfectant activity would increase permeation in cells and biofilms and kill more mycobacteria. Finally, aerosolized or waterborne mycobacteria may be trapped in filters coated with hydrophobic compounds (e.g., paraffin) and thereby intercepted before inhalation or ingestion.

## References

[1] Bernstein DI, Lummus ZL, Santilli G, Siskosky J, Bernstein IL. Machine operator’s lung. A hypersensitivity pneumonitis disorder associated with exposure to metalworking fluid aerosols. Chest. 1995;108:636–41. 10.1378/chest.108.3.636 7656609

[2] Embil J, Warren P, Yakrus M, Stark R, Corne S, Forrest D. Pulmonary illness associated with exposure to *Mycobacterium avium* complex in hot tub water: hypersensitivity pneumonitis or infection? Chest. 1997;111:813–6. 10.1378/chest.111.3.813 9118726

[3] Kahana LM, Kay JM, Yakrus M, Waserman S. *Mycobacterium avium* complex infection in an immunocompetent young adult related to hot tub exposure. Chest. 1997;111:242–5. 10.1378/chest.111.1.242 8996025

[4] Martyny JW, Rose CS. Nontuberculous mycobacterial bioaerosols from indoor warm water sources cause granulomatous lung disease. Indoor Air. 1999;9:1–6.

[5] Rose CS, Martyny JW, Newman LS, Milton DK, King TE Jr, Beebe JL, “Lifeguard lung”: endemic granulomatous pneumonitis in an indoor swimming pool. Am J Public Health. 1998;88:1795–800. 10.2105/AJPH.88.12.1795 9842376 PMC1509038

[6] Shelton BG, Flanders WD, Morris GK. *Mycobacterium* sp. as a possible cause of hypersensitivity pneumonitis in machine workers. Emerg Infect Dis. 1999;5:270–3. 10.3201/eid0502.990213 10221881 PMC2640702

[7] Muilenberg ML, Burge HT, Sweet T. Hypersensitivity pneumonitis and exposure to acid-fast bacilli in coolant aerosols. J Allergy Clin Immunol. 1998;91:311.

[8] Brunekreef B. Damp housing and adult respiratory symptoms. Allergy. 1992;47:498–502. 10.1111/j.1398-9995.1992.tb00672.x 1485653

[9] Andersson MA, Nikulin M, Koljalg U, Andersson MC, Rainey F, Reijula K, Bacteria, molds, and toxins in water-damaged building materials. Appl Environ Microbiol. 1997;63:387–92. 9023919 10.1128/aem.63.2.387-393.1997 PMC168331

[10] Eisen EA, Tolbert PE, Smith TJ, Monson RR, Hallock M, Woskie SR, Mortality studies of machining fluids; an exposure-response analysis of respiratory and digestive cancers. Proceedings of the 9th International Symposium on Epidemiology in Occupational Health, Sept 23-25, 1992, Cincinnati, Ohio. DHHS (NIOSH) pub no. 94-112. Cincinnati (OH): National Institute for Occupational Safety and Health; 1994. p. 113–7.

[11] Howell JK, Lucke WE, Steigerwald JC. Metalworking fluids: composition and use. The Industrial Metalworking Environment: Assessment and Control (Symposium). Nov. 13–16, 1995. Detroit: Automobile Manufacturers Association; 1996. p. 13–20.

[12] Centers for Disease Control and Prevention. Biopsy-confirmed hypersensitivity pneumonitis in automobile production workers exposed to metalworking fluids—Michigan, 1994–1995. MMWR Morb Mortal Wkly Rep. 1996;45:606–10. 8676853

[13] Centers for Disease Control and Prevention. Respiratory illness in workers exposed to metalworking fluid contaminated with nontuberculous mycobacteria—Ohio, 2001. MMWR Morb Mortal Wkly Rep. 2002;51:349–52. 12004986

[14] Wilson RW, Steingrube VA, Bottger EC, Springer B, Brown-Elliot BA, Vincent V, *Mycobacterium immunogenum* sp. nov., a novel species related to *Mycobacterium abscessus* and associated with clinical disease, pseudo-outbreaks and contaminated metalworking fluids: an international cooperative study on mycobacterial taxonomy. Int J Syst Evol Microbiol. 2001;51:1751–64. 11594606 10.1099/00207713-51-5-1751

[15] Moore JS, Christensen M, Wilson RW, Wallace RJ Jr, Zhang Y, Nash DR, Mycobacterial contamination of metal working fluids: involvement of a possible new taxon of rapidly growing mycobacteria. Am Ind Hyg Assoc J. 2000;61:205–13. 10.1202/0002-8894(2000)061<0205:MCOMFI>2.0.CO;2 10782192

[16] van Klingeren B, Pullen W. Comparative testing of disinfectants against *Mycobacterium tuberculosis* and *Mycobacterium terrae* in a quantitative suspension test. J Hosp Infect. 1987;10:292–8. 10.1016/0195-6701(87)90012-0 2891759

[17] Falkinham JO III, George KL, Parker BC, Gruft H. In vitro susceptibility of human and environmental isolates of *Mycobacterium avium, M. intracellulare*, and *M. scrofulaceum* to heavy-metal salts and oxyanions. Antimicrob Agents Chemother. 1984;25:137–9. 6230989 10.1128/aac.25.1.137 PMC185453

[18] Guerin WF, Jones GE. Mineralization of phenanthrene by a *Mycobacterium* sp. Appl Environ Microbiol. 1988;54:937–44. 3377503 10.1128/aem.54.4.937-944.1988 PMC202576

[19] Krulwich TA, Pelliccione NJ. Catabolic pathways of coryneforms, nocardias, and mycobacteria. Annu Rev Microbiol. 1979;33:95–111. 10.1146/annurev.mi.33.100179.000523 227323

[20] Combourieu B, Besse P, Sancelme M, Veschambre H, Delort AM, Poupin P, Morpholine degradation pathway of *Mycobacterium aurum* MO1: direct evidence of intermediates by in situ ^1^H nuclear magnetic resonance. Appl Environ Microbiol. 1998;64:153–8. 9435073 10.1128/aem.64.1.153-158.1998 PMC124686

[21] Falkinham JO III, Norton CD, Le Chevallier MW. Factors influencing numbers of *Mycobacterium avium, Mycobacterium intracellulare*, and other mycobacteria in drinking water distribution systems. Appl Environ Microbiol. 2001;67:1225–31. 10.1128/AEM.67.3.1225-1231.2001 11229914 PMC92717

[22] Schulze-Röbbecke R, Fischeder R. Mycobacteria in biofilms. Zentralbl Hyg Umweltmed. 1989;188:385–90. 2667558

[23] Havelaar AH, Berwald LG, Groothuis DG, Baas JG. Mycobacteria in semi-public swimming pools and whirlpools. Zentralbl Hyg Umweltmed. 1985;180:505–14. 4024777

[24] Mangione EJ, Huitt G, Lenaway D, Beebe J, Bailey A, Figoski M, Nontuberculous mycobacterial disease following hot tub exposure. Emerg Infect Dis. 2001;7:1039–42. 10.3201/eid0706.010623 11747738 PMC2631894

[25] Cirillo JD, Falkow S, Tompkins LS, Bermudez LE. Interaction of *Mycobacterium avium* with environmental amoebae enhances virulence. Infect Immun. 1997;65:3759–67. 9284149 10.1128/iai.65.9.3759-3767.1997 PMC175536

[26] Strahl ED, Gillaspy GE, Falkinham JO III. Fluorescent acid-fast microscopy for measuring phagocytosis of *Mycobacterium avium, Mycobacterium intracellulare*, and *Mycobacterium scrofulaceum* by *Tetrahymena pyriformis* and their intracellular growth. Appl Environ Microbiol. 2001;67:4432–9. 10.1128/AEM.67.10.4432-4439.2001 11571139 PMC93186

[27] Taylor RH, Falkinham JO III, Norton CD, LeChevallier MW. Chlorine, chloramine, chlorine dioxide, and ozone susceptibility of *Mycobacterium avium.* Appl Environ Microbiol. 2000;66:1702–5. 10.1128/AEM.66.4.1702-1705.2000 10742264 PMC92045

[28] Parker BC, Ford MA, Gruft H, Falkinham JO III. Epidemiology of infection by nontuberculous mycobacteria. IV. Preferential aerosolization of *Mycobacterium intracellulare* from natural water. Am Rev Respir Dis. 1983;128:652–6. 6354024 10.1164/arrd.1983.128.4.652

[29] Schulze-Röbbecke R, Buchholtz K. Heat susceptibility of aquatic mycobacteria. Appl Environ Microbiol. 1992;58:1869–73. 1622262 10.1128/aem.58.6.1869-1873.1992 PMC195697

[30] duMoulin GC, Stottmeier KD, Pelletier PA, Tsang AY, Hedley-Whyte J. Concentration of *Mycobacterium avium* by hospital hot water systems. JAMA. 1988;260:1599–601. 10.1001/jama.260.11.1599 3411741

[31] Hüttunen K, Ruotsalainen M, Iivanainen E, Torkko P, Katila M-L, Hirvonen M-R. Inflammatory responses in RAW264.7 macrophages caused by mycobacteria isolated from moldy houses. Environ Toxicol Pharmacol. 2000;8:237–44. 10.1016/S1382-6689(00)00047-8 10996543

[32] Rha Y-H, Taube C, Haczku A, Joetham A, Takeda K, Duez C, Effect of microbial heat shock proteins on airway inflammation and hyperresponsiveness. J Immunol. 2002;169:5300–7. 12391250 10.4049/jimmunol.169.9.5300

[33] Wendt SL, George KL, Parker BC, Gruft H, Falkinham JO III. Epidemiology of infection by nontuberculous mycobacteria. III. Isolation of potentially pathogenic mycobacteria in aerosols. Am Rev Respir Dis. 1980;122:259–63. 7416602 10.1164/arrd.1980.122.2.259

[34] George KL, Parker BC, Gruft H, Falkinham JO III. Epidemiology of infection by nontuberculous mycobacteria. II. Growth and survival in natural waters. Am Rev Respir Dis. 1980;122:89–94. 7406344 10.1164/arrd.1980.122.1.89

[35] Brennan PJ, Nikaido H. The envelope of mycobacteria. Annu Rev Biochem. 1995;64:29–63. 10.1146/annurev.bi.64.070195.000333 7574484

[36] Collins FM. Bactericidal activity of alkaline glutaraldehyde solution against a number of atypical mycobacterial species. J Appl Bacteriol. 1986;61:247–51. 3096931 10.1111/j.1365-2672.1986.tb04283.x

[37] George KL, Pringle AT, Falkinham JO III. The cell surface of *Mycobacterium avium – intracellulare* and *M. scrofulaceum*: effect of specific chemical modifications on cell surface charge. Microbios. 1986;45:199–207. 3736439

[38] Young H-K. Antimicrobial resistance spread in aquatic environments. J Antimicrob Chemother. 1993;31:627–35. 10.1093/jac/31.5.627 8335494

[39] Wolinsky E. Nontuberculous mycobacteria and associated diseases. Am Rev Respir Dis. 1979;119:107–59. 369415 10.1164/arrd.1979.119.1.107

[40] Clark-Curtiss JE. Identification of virulence determinants in pathogenic mycobacteria. Curr Top Microbiol Immunol. 1998;225:57–121. 9386328 10.1007/978-3-642-80451-9_4

[41] Speizer FE. Occupational and environmental lung diseases: an overview. Environ Health Perspect. 2000;108(Suppl 4):603–4. 10931778 10.1289/ehp.00108s4603 PMC1637669

